# Side Effects: Substantial Non-Neutral Evolution Flanking Regulatory Sites

**DOI:** 10.1371/journal.pgen.1003528

**Published:** 2013-05-30

**Authors:** James G. D. Prendergast, Colin A. Semple

**Affiliations:** MRC Institute of Genetics and Molecular Medicine, University of Edinburgh, Western General Hospital, Edinburgh, United Kingdom; Stanford University School of Medicine, United States of America

In the pre-genome era, most of what we knew about molecular evolution could be traced to our knowledge of the genetic code, and the impact of DNA sequence variation on protein structure and, by inference, protein function [1]. But in the post-genome era, it has become clear that the fraction of functional sequence—estimated using comparative approaches to identify residues that “escape” genetic drift—far exceeds the fraction explained by protein-coding genes. In mammals, somewhere between 5% and 15% of the genome is evolutionarily constrained, and is presumably functional [2]. *Drosophila* and other invertebrate genomes may have much larger proportions (47%–70%) of constrained nucleotides [3]; in all cases, the proportions of nucleotides found to be conserved dwarves those encoding proteins (around 1% in humans and 20% in flies)—which prompts the question: what aspects of genomic function might explain these apparent excesses of conserved sequence?

Over the past few years, it has become clear that the physical organization and structure of the genome within cells, over a range of scales, also casts discernable shadows on the sequence. This is the realm of chromatin structure (the many combinations of proteins associated with the DNA), which adopts an undulating landscape along chromosomes associated with cellular functions such as transcription. The binding of a range of proteins to eukaryotic genomes has been shown to be linked to variation in the underlying DNA sequence. The specific regions of the human genome known to be bound by transcription factors often display remarkable patterns of conservation that parallel the structure of the DNA-binding interface of the protein involved [4]. More broadly, characteristic fluctuations in sequence divergence have been observed corresponding to nucleosome cores and intervening linker sequences across a variety of species [5], and there is evidence that this reflects the action of selection [6]. However, recent data from the ENCODE Consortium has suggested that perhaps 80% of the human genome is functional, in the sense that it is subject to a biochemical modification in at least one cell type [7]. This substantially exceeds all estimates of the proportion of human nucleotides under constraint, including those used by the ENCODE Consortium [8], and the discrepancy has led to some notably animated discussion [9]. There is therefore a large gap between the proportion of the genome thought to be functional via evolutionary studies and the proportion that appears functional, according to the presence of particular chromatin features. This gap also appears to exist, though to a lesser extent, in *Drosophila*, where over 90% of the genome has been assigned a biochemical role of some description [10]. In this issue of *PLOS Genetics*, Kenigsberg and Tanay [11] have investigated the links between chromatin and sequence evolution from the point of view of conserved noncoding elements (CNEs), and may have found a way to begin to bridge the gap. Rather than examining DNA sequence conservation at the sites of a particular chromatin state, they have investigated the characteristics of CNEs in the *Drosophila* genome, within their genomic and chromatin context.

**Figure 1:**
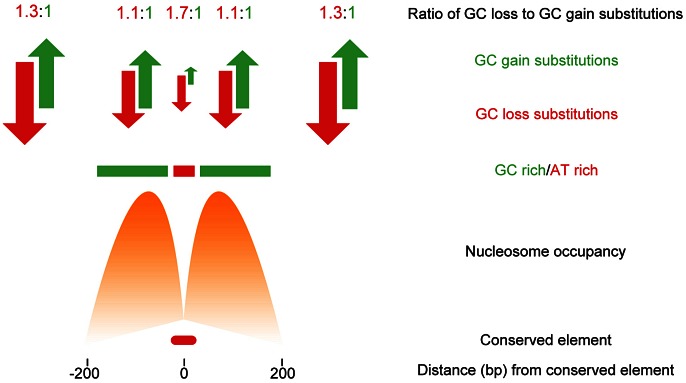
Non-neutral evolution within large regions flanking CNEs in *Drosophila* acts to maintain sequence composition and favourable nucleosome positioning.

Kenigsberg and Tanay first identified approximately 68,000 short (mean length of 50 bp) regions of the genome whose rate of divergence was at least two times lower than expected. These CNEs were observed to coincide with the location of a range of chromatin features, suggesting underlying DNA sequence conservation is a feature of a range of functional chromatin states in *Drosophila*. Although these CNEs covered only around 3% of the *Drosophila* genome, they were found to have characteristic sequence compositional biases. The vast majority of these short elements were centered upon a small (20–30 bp), unusually AT rich, focal region. However, it was found these short AT rich regions were embedded in larger (several hundred base pairs), relatively GC rich regions. Surprisingly, these patterns were observed at CNEs irrespective of the functional chromatin state seen at the CNE, including states associated with promoters, enhancers, repressed sites, and insulator sites. Compositional biases have previously been noted as a common feature of some regulatory sites, and this study shows these compositional biases are linked to the positioning of nucleosomes on either side of such sites. Nucleosomes have been shown to preferentially associate with GC rich regions of DNA, and, in species from yeast to humans, nucleosome positioning appears to be maintained by a balance in the number of A/T relative to G/C base pair gaining substitutions maintained by selection [6], [12]. Kenigsberg and Tanay report a similar balance in the gain and loss of GC dinucleotides, maintaining elevated GC content on either side of the relatively AT rich *Drosophila* CNEs, and suggest this balance is also likely to be maintained by selection. They conclude that although only a small proportion of the genome (within CNEs) displays evidence for strong evolutionary constraint, a substantially larger proportion, approximately 25%, is evolving non-neutrally due to the milder selective constraints imposed to maintain the surrounding local chromatin structure (Figure 1). This raises the possibility that large swathes of any genome may be subject to rather modest, and often elusive, levels of constraint on sequence composition as an extended side effect of the presence of neighbouring regulatory sites.

Kenigsberg and Tanay go on to show that the rate of base substitutions, as measured by population polymorphisms, is also dependent on the base composition of the region considered. For instance, GC depleting substitutions were observed to be underrepresented at GC rich regions. This was found to be the case not only in flies, but also when mouse and human data were examined. Together, these data suggest that structural constraints are impacting the evolutionary dynamics of current populations across a range of eukaryotic organisms. They also support a new worldview in evolutionary genomics, where a complete understanding of sequence variation and its effects on function is only possible by considering the genome as a physical molecule. Genome evolution may be seen more clearly seen through the lens of the epigenome.
